# Pathobiology of highly pathogenic H5 avian influenza viruses in naturally infected Galliformes and Anseriformes in France during winter 2015–2016

**DOI:** 10.1186/s13567-022-01028-x

**Published:** 2022-02-14

**Authors:** Nicolas Gaide, Marie-Noëlle Lucas, Mattias Delpont, Guillaume Croville, Kim M. Bouwman, Andreas Papanikolaou, Roosmarijn van der Woude, Iwan A. Gagarinov, Geert-Jan Boons, Robert P. De Vries, Romain Volmer, Angélique Teillaud, Timothée Vergne, Céline Bleuart, Guillaume Le Loc’h, Maxence Delverdier, Jean-Luc Guérin

**Affiliations:** 1 IHAP, Toulouse University, ENVT, INRAE, Toulouse, France; 2 Department of Pathobiology, Faculty of Veterinary Medicine, Utrecht University, Utrecht, The Netherlands; 3 Department of Chemical Biology & Drug Discovery, Utrecht Institute for Pharmaceutical Sciences, Utrecht University, 3584 CG Utrecht, The Netherlands; 4 Bijvoet Center for Biomolecular Research, Utrecht University, Utrecht, The Netherlands; 5 Complex Carbohydrate Research Center, University of Georgia, 315 Riverbend Rd, Athens, GA 30602 USA

**Keywords:** Highly pathogenic avian influenza, viral tropism, H5, pathology, sialic acids

## Abstract

In late 2015, an epizootic of Highly Pathogenic Avian Influenza (H5Nx) was registered in Southwestern France, including more than 70 outbreaks in commercial poultry flocks. Phylogenetic analyses suggested local emergence of H5 viruses which differed from A/goose/Guangdong/1/1996 clade 2.3.4.4b lineage and shared a unique polybasic cleavage site in their hemagglutinin protein. The present work provides an overview of the pathobiological picture associated with this epizootic in naturally infected chickens, guinea fowls and ducks. Upon necropsy examination, selected tissues were sampled for histopathology, immunohistochemistry and quantitative Real Time Polymerase Chain Reaction. In Galliformes, HPAIVs infection manifested as severe acute systemic vasculitis and parenchymal necrosis and was associated with endothelial expression of viral antigen. In ducks, lesions were mild and infrequent, with sparse antigenic detection in respiratory and digestive mucosae and leukocytes. Tissue quantifications of viral antigen and RNA were higher in chickens and guinea fowls compared to duck. Subsequently, recombinant HA (rHA) was generated from a H5 HPAIV isolated from an infected duck to investigate its glycan-binding affinity for avian mucosae. Glycan-binding analysis revealed strong affinity of rHA for 3’Sialyl-LacNAc and low affinity for Sialyl-Lewis^X^, consistent with a duck-adapted virus similar to A/Duck/Mongolia/54/2001 (H5N2). K222R and S227R mutations on rHA sequence shifted affinity towards Sialyl-Lewis^X^ and led to an increased affinity for chicken mucosa, confirming the involvement of these two mutations in the glycan-binding specificity of the HA. Interestingly, the rHA glycan binding pattern of guinea fowl appeared intermediate between duck and chicken. The present study presents a unique pathobiological description of the H5 HPAIVs outbreaks that occurred in 2015–2016 in Southwestern France.

**Supplementary Information:**

The online version contains supplementary material available at 10.1186/s13567-022-01028-x.

## Introduction

Avian influenza is a highly contagious infectious disease caused by Influenza A viruses belonging to the *Orthomyxoviridae* family [1]. Wild birds represent the natural reservoir contributing to viral spread and generation of occasional epizootics characterized by massive die-offs of both wild and domestic birds, as well as significant economic losses [2, 3].

Between November 2015 and January 2016, an epizootic of Highly Pathogenic Avian Influenza (HPAI) H5 (N1, N2 and N9) was registered in commercial poultry flocks in France, with more than 70 confirmed cases distributed in the South-West of the country [4]. Free-range, mule duck farms, dedicated to foie-gras production, comprised more than 80% of identified cases, while 20% of cases were registered in chicken and guinea fowl flocks. Ducks appeared mostly asymptomatic while Galliformes presented a mild increase in mortality [5]. Genetic analysis revealed that the HPAI viruses isolated from infected birds belonged to a monophylogenic group of Eurasian lineage and shared the same original hemagglutin cleavage site (HQRRKR**/**GLF). These findings suggested the emergence of different H5Nx reassortants from a locally circulating H5 AIV ancestor [4].

Viral tropism is defined as the ability of viral strains to infect different types of cells, organs and hosts [6]. It results from the combination of both viral and host genotypes, defining the modalities of this interaction such as restriction to target cell (*accessibility*), receptor-dependent entry into host cells (*cellular susceptibility*), replication cycle completion of the replication cycle and release of the viral progeny (*permissiveness*) [7–9]. Susceptibility represents one of the most studied determinant of AIVs tropism, and the surveillance of receptors specificity appears crucial to track interspecies barrier crossing and zoonotic transmission. AIVs are known to preferentially bind to α2–3 linked *N*-acetylneuraminic sialic acid (SA) residues of glycoproteins through the hemagglutin protein, while human adapted-influenza viruses preferentially bind to α2–6 SAs [10]. Pigs and minor poultry species, the Japanese quail in particular, possess both α2–3 and α2–6 SAs and represent potential mixing vessels providing a suitable environment for AIV receptor switching [11].

Among avian species, other structural determinants of the hemagglutinin protein contribute to species restrictions by modulating glycan binding specificity. Chicken and duck-adapted AIVs exhibit binding preferences for fucosylated α2–3 SAs (Sialyl-Lewis^X^) and non-fucosylated α2–3 SAs (3’Sialyl-LacNAc), respectively [13, 14]. Studies on Eurasian H5 HP and LPAIVs suggested that two amino acid residues in the primary sequence of the hemagglutin protein located at the positions 222 and 227 are involved in those affinity patterns. K222 and S227 were highly conserved among duck-adapted AIVs, while K222R and S227R substitutions were observed in chicken-adapted AIVs and appeared sufficient to shift SAs specificity towards Sialyl-Lewis^X^, and promote adaptation to galliformes species [12, 13].

The present study aims to characterize the pathobiology of naturally infected Galliformes and Anseriformes originating from H5 HPAIV reassortants that emerged locally in France during winter 2015–2016. For this purpose, we used a multidisciplinary approach combining necropsy, histopathology, immunohistochemistry, molecular biology and glycobiology assays. To the best of our knowledge, these data represent a unique pathological description of this epizootic and provide new pathological data on naturally infected guinea fowls, a species in which HPAIV infection is still poorly documented.

## Materials and methods

### Field cases sampling

A total of 9 commercial poultry flocks were included in the study, including 4 broiler chickens, 3 guinea fowls and 2 mule duck flocks (Additional file 1). Following official procedures, all flocks were tested positive for H5 HPAIV by RT-PCR at the French National Laboratory for Avian Influenza and Newcastle disease, between December 2015 and January 2016 [14]. The phylogenetic relationship between H5 HPAIVs isolates from this epizootic has been previously reported [4]. Additional phylogenetic analysis is provided in the Additional file 2, based on hemagglutinin sequences from 7 H5 HPAIV isolates from guinea fowls, ducks and chickens. For each flock, a complete necropsy was performed on clinically affected birds (dead or euthanized) for a total of 35 individuals (16 chickens, 9 guinea fowls and 10 ducks) and macroscopic lesions were recorded. Subsequently, sections of brain, trachea, lung, heart, liver, pancreas, spleen, proventriculus, large intestine (ceca) and kidney were collected and fixed in 10% neutral buffered formalin to assess the presence of lesions and investigate viral tropism. Concurrently, fresh tissue sections of brain, lung, spleen and intestine, obtained from a total of 38 birds (16 chickens, 12 guinea fowls and 10 ducks) were stored at −80 °C for viral detection by molecular biology. On-farm investigations and collection of samples were performed in strict compliance with regulations and biosecurity procedures, with the authorization and supervision of official veterinary services, and before the implementation of depopulation and carcass disposal.

### Histopathology and immunohistochemistry

Formalin-fixed tissues were routinely processed for the production of paraffin blocks, sectioned at 4 μm, stained with hematoxylin and eosin (HE) and examined by light microscopy. Immunostaining was performed on paraffin-embedded tissues using a monoclonal mouse antibody against Influenza A virus nucleoprotein (NP, Biomérieux, France, 11-030). Specifically, the immunohistochemical protocol included an antigen retrieval step with pronase 0.05% applied for 10 min at 37 °C, a peroxidase blocking step of 5 min at room temperature (Dako, Glostrup, Denmark, S2023), followed by saturation of non-specific binding sites with normal goat serum (Dako, Glostrup, Denmark, X0907) for 25 min at room temperature. Finally, overnight incubation was conducted at 4 °C with anti-NP antibody at 1:50 dilution. Immunohistochemical labelling was revealed with a biotinylated polyclonal goat anti-mouse immunoglobulin conjugated with horseradish peroxidase (HRP) antibody (Dako, Glostrup, Denmark, LSAB2 system-HRP, K0675) and the 3,3′-Diaminobenzidine (DAB) chromogen of the HRP (Dako, Glostrup, Denmark, DAB Substrate-Chromogen, K3468). Negative controls included sections incubated either without the specific primary antibody or with another monoclonal antibody of the same isotype (IgG2) for the three avian species included in this study.

Prior to the study, histopathological diagnoses were conducted by two veterinary pathologists certified by the European College of Veterinary Pathology (ECVP). An additional ECVP pathologist performed frequency and scoring assessments on all samples. For this purpose, 4-point scale scoring systems were applied to compare both histological lesions and viral antigenic biodistribution (Additional files 3 and 4).

To confirm inter-species differences observed in lung and spleen through a semi-quantitative histopathological and immunohistochemistry scoring, a computer-assisted quantification of nucleoprotein-positive cell density was determined on immunohistochemical sections using the NuclearQuant® software (3DHISTECH, France). For each tissue, slides were scanned at 40 × magnification with a Panoramic Slide Scan (3DHISTECH, France). Nuclear detection and positivity thresholds were first assessed with a negative and a positive control slide. Analysis was then assessed for each tissue on a representative region of interest of 6.4 ± 1.5 mm2. Results were expressed as NP positive cells per mm2 of tissue.

### RNA extraction and qRT-PCR

All samples were processed in a BSL3 laboratory until the completion of the lysis step, in strict compliance with biosafety procedures. For RNA extraction, swabs were placed in 500 µL of 1X PBS, vortexed for 30 s, while 30–60 mg of each tissue were placed in 200 µL PBS 1X containing 0.8 µg/µL of proteinase K (Thermo fisher #EO0492) and incubated for 20 min at 37 °C. For all tissue samples, RNA was extracted from 140 µL of supernatant with QIAamp® Viral RNA Mini Kit (QIAGEN #52,906) and then kept at −80 °C. RT-qPCR was performed on 2 µL RNA using iTaq™ Universal SYBR® Green Supermix (Bio-Rad #172–5125). M and H5 genes were targeted with M52C / M253R primers for M gene [15], and H5_HP_EA_F2 (5′-TCCTTGCAACAGGACTAAG-3′)/H5_HP_EA_R (5′-GTCTACCATTCCYTGCCA-3′) [16]. Absolute quantification was performed with a plasmid range from 10^2^ to 10^7^ copies/reaction (2 µL). RTqPCR reaction and results interpretation were performed on a LightCycler 96 instrument (Roche). Results were expressed as viral RNA copy number per mg of tissue.

### Expression and purification of HA for binding studies

For glycan and tissue binding assays, H5-encoding cDNAs from a duck isolate A/duck/France/150236/15 (H5N9), A/Chicken/Ibaraki/ (H5N2, IBR), A/Hong Kong/483/97 (H5N1) and A/PR/8/34 (H1N1, Cambrigde strain) were cloned into the pCD5 expression vector as previously described [17]. Briefly, expression vectors included DNA sequences coding for a signal sequence, a GCN4 trimerization motif (RMKQIEDKIEEIESKQKKIENEIARIKK), and Strep-tag II (WSHPQFEK; IBA, Germany). HA proteins cell were expressed on transfected HEK293S GnTI (-), produced and purified as previousy described [17].

### Glycan microarray binding of HA

Purified, soluble trimeric HA was precomplexed with an anti-Strep-tag mouse antibody and Alexa 647-linked anti-mouse IgG (4:2:1 molar ratio) prior to incubation for 15 min on ice in 100 μL phosphate-buffered saline (PBS)–Tween (PBS-T) and incubated on the array surface in a humidified chamber for 90 min. Slides were subsequently washed by successive rinses with PBS-T, PBS, and deionized H_2_O. Washed arrays were dried by centrifugation and immediately scanned. The slides were scanned using an Innopsys Innoscan 710 microarray scanner at the appropriate excitation wavelength. To ensure that all signals were in the linear range of the scanner’s detector and to avoid any saturation of the signals various gains and photomultiplier tubes (PMT) values were employed. Images were analyzed with Mapix software (version 8.1.0 Innopsys) and processed with our home written Excel macro. The average fluorescence intensity and SD were measured for each compound after exclusion of the highest and lowest intensities from the spot replicates (*n* = 4).

### Tissue microarray binding of HA

To investigate the mucosal susceptibility of chickens, guinea fowls and ducks to the H5 HPAIV involved in the epizootic, binding affinity of wildtype H5 recombinant hemagglutin (H5 rHA) was determined on tissue microarrays obtained from each species of interest, and compared to the chicken-adapted IBR H5N2 rHA. Additionally, 222 and 227 mutated versions of both rHAs were generated to switch affinity from duck to chicken, for H5N9 rHA, and from chicken to duck, for IBR H5N2 rHA, according to Hiono et al. [13]. Serial sections of formalin-fixed, paraffin-embedded tissues were obtained from the Department of Veterinary Pathobiology, Faculty of Veterinary Medicine, Utrecht University, The Netherlands. Selected tissues included trachea and colon (*n* = 1 bird per tissue) obtained from guinea fowl, chicken and several duck breeds [18]. Tissue sections were rehydrated in a series of alcohol solutions (100%, 96% and 70%) and lastly in distilled water. For antigen retrieval, slides were boiled in citrate buffer pH 6.0 for 10 min at 900 kW in a microwave and washed in PBS-T three times. Endogenous peroxidase activity was blocked with 1% hydrogen peroxide applied for 30 min. Tissues were subsequently incubated with 3% BSA in PBS-T overnight at 4 °C. The next day, purified, soluble trimeric HA was precomplexed with an anti-Strep-tag mouse antibody and Alexa 647-linked anti-mouse IgG (4:2:1 molar ratio) prior to incubation for 15 min on ice in 100 μL phosphate-buffered saline (PBS)–Tween (PBS-T) and incubated on the array surface in a humidified chamber for 90 min. After staining, slides were washed three times with PBS and AEC was applied for 15 min. Slides were then washed with water for 5 min, counterstained with hematoxylin, washed with water for 10 min, dried and finally mounted with aquatex (Sigma-Aldrich, St. Louis, Missouri, USA).

### Statistical analysis

Non-parametric Kruskal–Wallis test with Dunn-Bonferroni post hoc tests were used to compare histopathological scoring and RNA viral load (qRT-PCR) between guinea fowls, chickens and ducks. The average number of cells expressing the nucleoprotein per mm2 was measured using a computer-assisted nucleoprotein positive cell counting algorithm (QuantCenter, 3DHISTECH). For NP positive cell counting, a negative binomial regression model was applied using the R software [19]. The model was used to identify potential associations between the number of positive cells (response variable) and a selection of predictor variables, including the tissue (spleen and lung) and the host species (chicken, guinea fowl, duck). Likelihood-ratio tests were used to identify statistically significant variables with a significant *p*-value of less than 0.05.

## Results

### Necropsy findings

**Figure 1:**
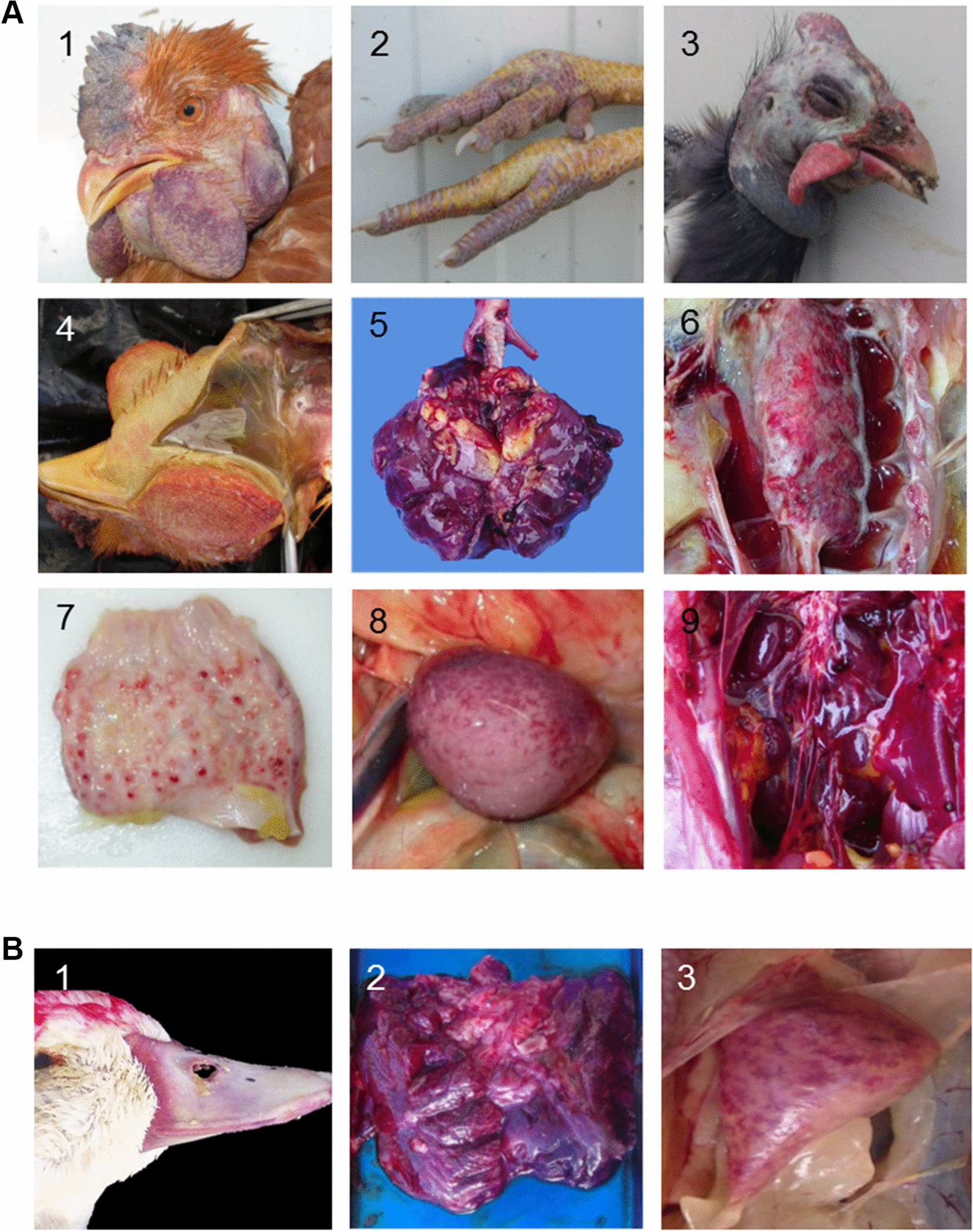
**Gross lesions of H5 HPAIV in naturally infected chickens, guinea fowls (A), and ducks (B)**. **A** 1, Facial edema, hemorrhages and necrosis involving comb, wattles and unfeathered skin, chicken. 2, Extensive cutaneous hemorrhages on legs, chicken. 3, Facial edema, guinea fowls. 4, Severe subcutaneous edema, chicken. 5, Diffuse congestion and edema, lung, guinea fowl. 6, Excess fluid within the lumina of air sacs, chicken. 7, Proventricular congestion and hemorrhages at the tip of proventricular glands, chicken 8, Hypertrophic and hemorrhagic appearance of spleen, chicken. 9, Hypertrophic and red appearance of kidney, chicken. **B** 1, Beak heterogeneous in color, with peripheral pallor and redness at the base, duck; 2, Diffuse congestion and edema, lung, duck. 3, Mottled and hypertrophic spleen, duck.

Gross lesions were common and severe in chickens and guinea fowls, but rare and mild in ducks. Upon external examination, facial subcutaneous edema, extending to comb, wattles and neck, was observed mainly in chickens (3/4 flocks) and guinea fowls (3/3 flocks) (Figure 1A). In chickens, cutaneous necrosis and subcutaneous hemorrhages involving face, abdomen and legs were occasionally present (3/4 flocks). In ducks, the external examination was unremarkable. Beaks were occasionally heterogeneous in color, with peripheral pallor and redness at the base (Figure 1B).

In all species, the most consistent changes included congested and edematous lungs and enlarged and mottled spleen (Figure 1). Ascites was occasionally identified in chickens and ducks. A few chickens presented proventricular glandular congestion and hemorrhages (Figure 1A). In ducks, visceral gross findings were rare, but included exudative airsacculitis, splenomegaly and visceral petechiae.

### Histopathological findings and viral antigen detection

**Figure 2:**
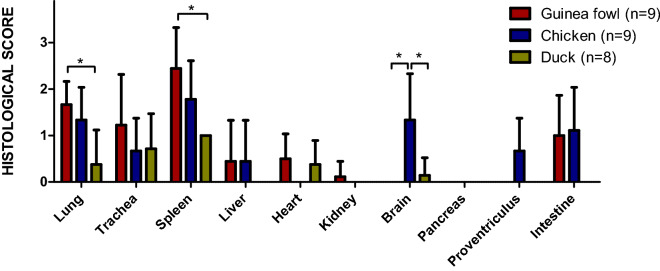
**Histopathological scoring comparison between naturally infected H5Nx guinea fowls, chicken and ducks.** Each bar represents mean with standard deviation (SD). * indicates statistical significance (*p* < 0.05, Kruskal–Wallis’ test with Dunn-Bonferroni post hoc test). Guinea Fowl (*n* = 9), Chicken (*n* = 16), Duck (*n* = 10, spleen *n* = 6 and intestine *n* = 4).

**Figure 3:**
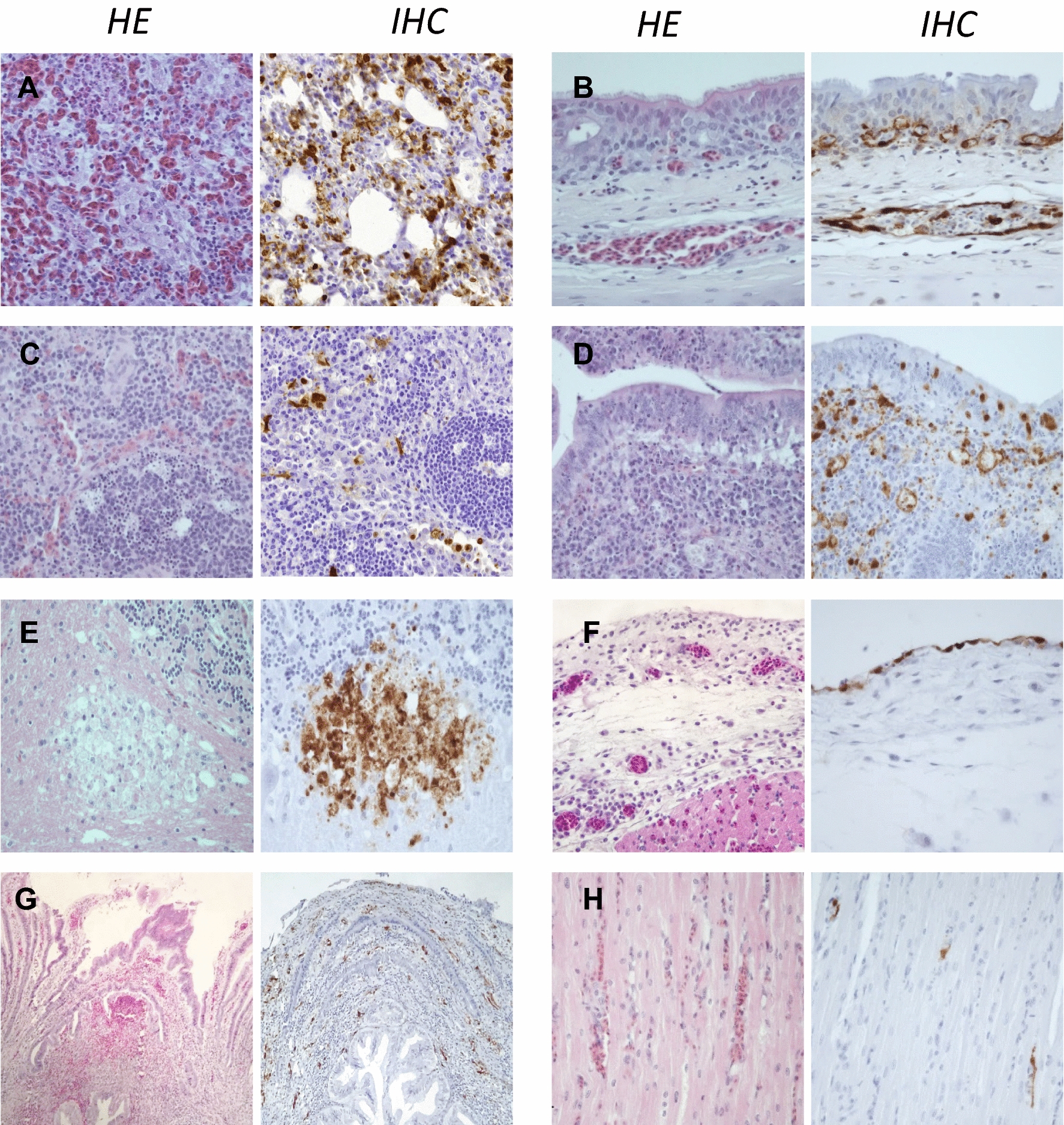
**Histopathological findings and viral antigen detection in H5 HPAIV naturally infected chickens. A** Lung, mixed leukocytic interstitial pneumonia. Frequent NP-positive leukocytes and endothelial cells in air capillaries. **B** Trachea, lamina propria congestion. Frequent NP-positive cells in endothelial cells. **C** Spleen, lymphocytolysis, single cell necrosis/apoptosis and mixed leukocytic infiltration. Frequent NP-positive leukocytes. **D** Intestine, lamina propria single cells necrosis/apoptosis, lymphocytolysis. Frequent NP-positive endothelial cells, leukocytes, epithelial lining and necrotic cell. **E** Brain, cerebellar foci of lytic necrosis. Abundant viral antigen staining within necrotic debris. **F** Epicardium, congestivo-edematous epicarditis with lymphocytic and macrophagic infiltration. Frequent NP-positive mesothelial cells. **G** Proventriculus, acute hemorrhages and congestion at the tip of a proventricular gland close to the primary duct. Frequent NP-positive endothelial cells. **H** Myocardium, congestion and mild heterophilic infiltration. Rare NP-positive endothelial cells. Hematoxylin and eosin (HE). Anti-nucleoprotein immunohistochemistry (IHC).

**Figure 4:**
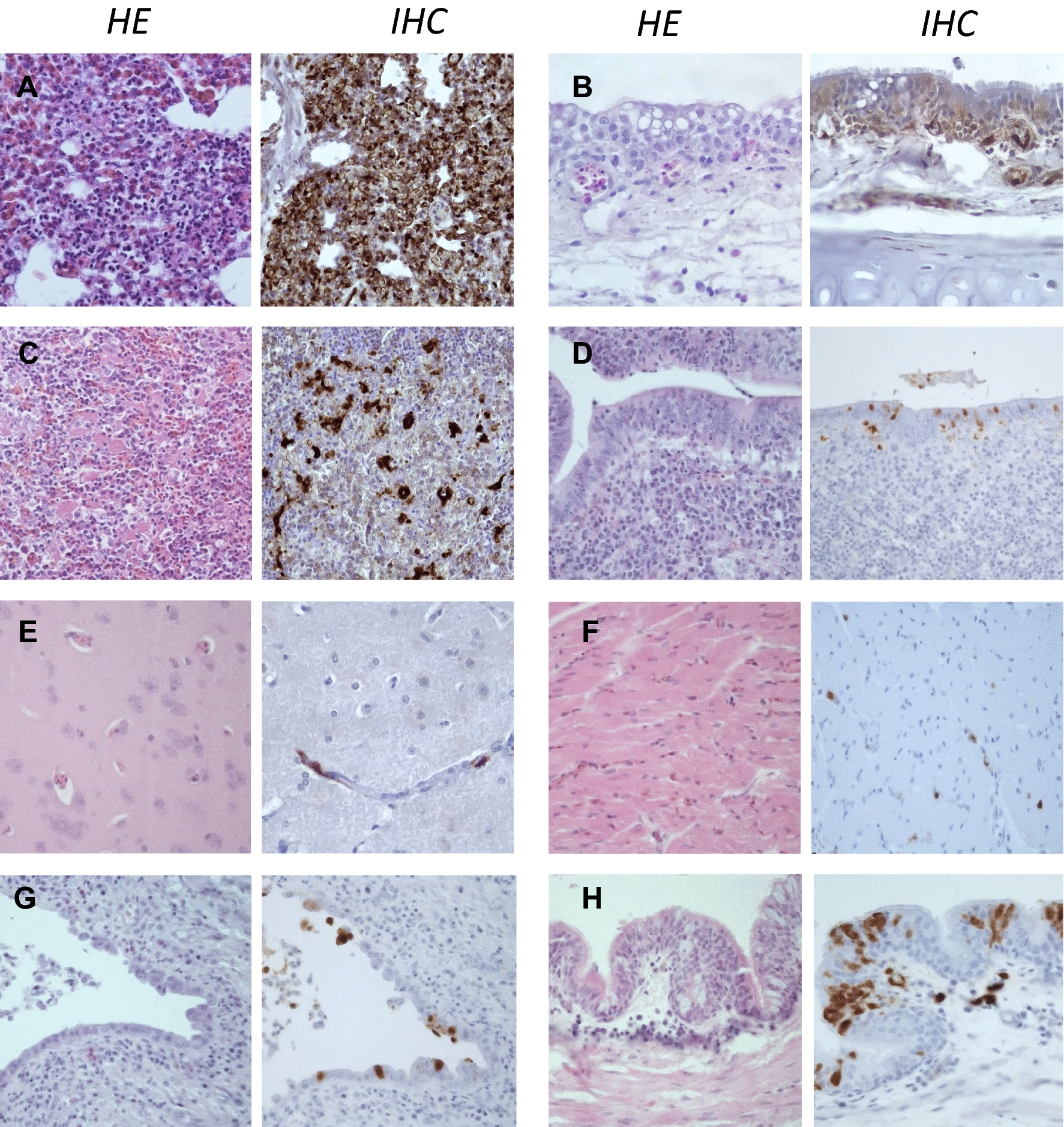
**Histopathological findings and viral antigen detection in H5 HPAIV naturally infected guinea fowls. A** Lung, mixed leukocytic interstitial pneumonia. Widespread NP-positive cells expanding air capillaries. **B** Trachea, epithelial degeneration and congestion. Widespread NP-positive cells in endothelial and epithelial cells. **C** Spleen, necrotizing splenitis with thrombosis. Widespread NP-positive endothelial cells and leukocytes. **D**, Cecum, disseminated single cells necrosis/apoptosis within lamina propria and epithelium. Frequent NP-positive cells within epithelium. **E**, Brain, within normal limits. Frequent NP-positive endothelial cells. **F** Heart, mild congestion, mild heterophilic infiltration. Sparse NP-positive cells in endothelial cells. **G** Liver bile duct, cholangiocellular degeneration and necrosis with sloughing and luminal cellular debris. Frequent multifocal viral antigen staining in epithelial cells of bile ducts and within luminal necrotic debris. **H** Bronchus, lymphoplasmacytic infiltration of lamina propria. Abundant NP-positive endothelial, epithelials cells and leukocytes. Hematoxylin and eosin (HE). Anti-nucleoprotein immunohistochemistry (IHC).

**Figure 5:**
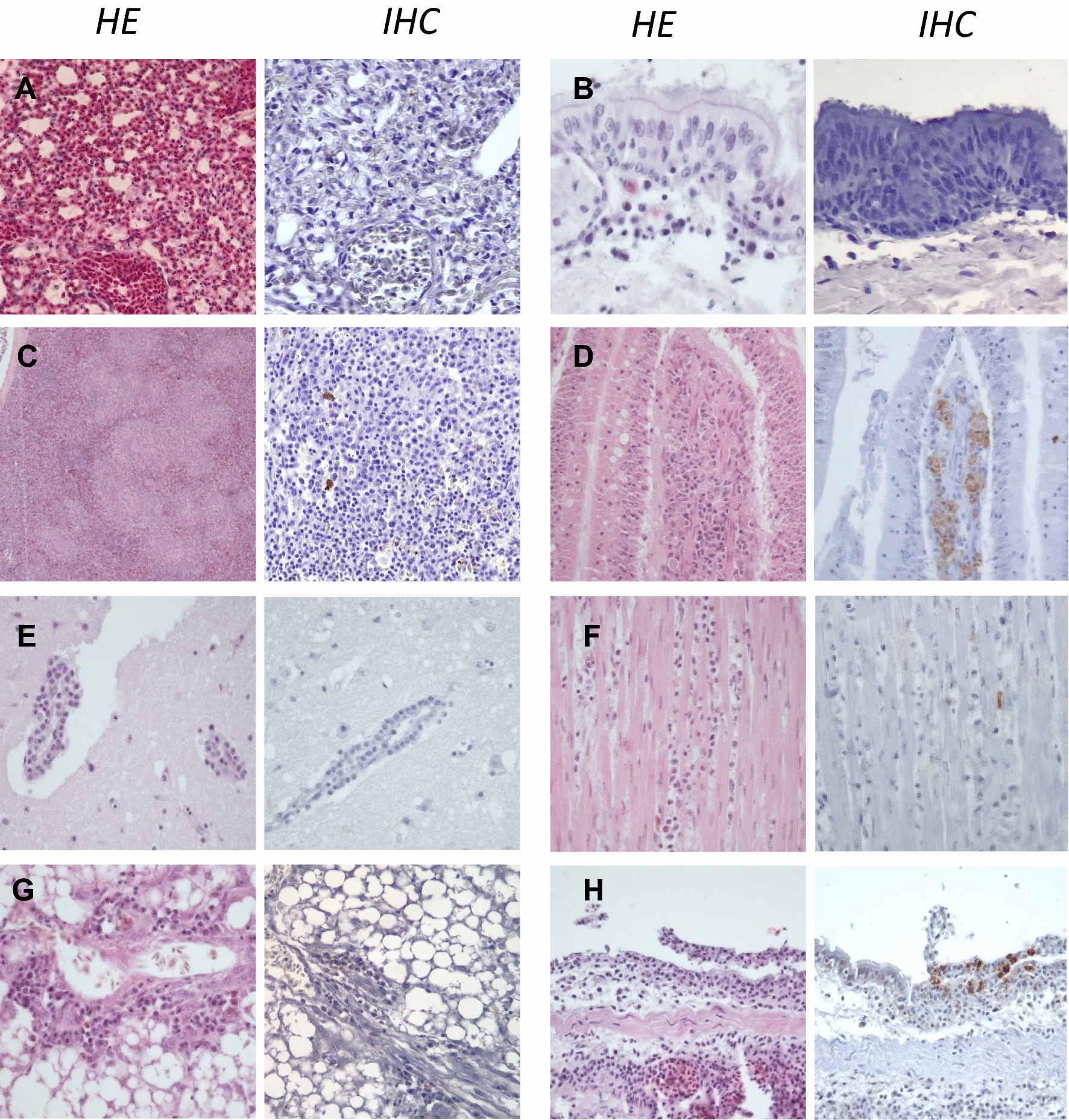
**Histopathological findings and viral antigen detection in H5 HPAIV naturally infected ducks**. **A** Lung, non-specific diffuse congestion. Absence of NP positive cells. **B** Trachea, lymphoplasmacytic infiltration of lamina propria. Absence of NP positive cells. **C** Spleen, macrophagic infiltration, lymphoid and reticular cell hyperplasia. Rare NP-positive leukocytes. **D** Intestine, lympho-plasmacytic infiltration of lamina propria. Sparse NP-cells within lamina propria, presumably leukocytes. **E** Brain, non suppurative encephalitis (observed in one subject). Absence of NP positive cells. **F** Heart, myocardial lympho-plasmacytic infiltration. Rare NP-positive cells within interstitium. **G** Liver, within normal limits with non-specific portal infiltration. Absence of NP positive cells. **H** Bronchus, necrotizing bronchitis with lymphoplasmacytic infiltration and congestion (observed in one subject). Frequent NP-Positive epithelial cells within mucosa and luminal debris. Hematoxylin and eosin (HE). Anti-nucleoprotein immunohistochemistry (IHC).

Splenic lesions were identified in 100% of individuals and species examined (Table 1). The severity score was significantly higher for guinea fowls compared to ducks (Figure 2). In Galliformes, lesions included congestion, heterophilic infiltration and fibrinoid necrosis of blood vessels with thrombosis and hemorrhages (Figures 3 and 4). In chickens, additional lymphocytolysis was frequently observed. In ducks, diffuse lymphoid and reticular cell hyperplasia was noticed. Nucleoprotein expression appeared widespread in guinea fowls and frequent but variable in chickens, with a strong endothelial distribution. In ducks, nucleoprotein positive cells, presumably leukocytes, were sparse and inconstant (Figure 5).

**Table 1:** **Histopathological and Immunohistochemical scores and distribution in naturally infected guinea fowls, chickens and ducks infected with H5 HPAIVs**

	Histopathology (lesion frequency and average score*)			Immunohistochemistry** (average intensity and distribution)					
Species *Dead* *Euthanized*	Guinea fowl *n* = *9* *–*	Chicken *n* = *9* *n* = *7*	Duck *n* = *8* *n* = *2*	Guinea fowl		Chicken		Duck	
Lung	100% (1.7) –	89% (1.3) 100% (1.3)	25% (0.4) 100% (1.0)	+++	*Air capillaries, endothelial cells, leukocytes*	++	*Air capillaries, endothelial cells*	+	*Leukocytes, epithelial bronchial cells*
Trachea	67% (1.2) –	56% (0.7) 86% (1.0)	57% (0.7) 0% (0)	+++	*Endothelial cells, epithelial cells*	++	*Endothelial cells*	+	*Epithelium and necrotic debris*
Spleen	100% (2.4) –	100% (1.8) 100% (1.7)	100% (1.0) 100% (1.0)	+++	*endothelial cells, leukocytes*	++	*endothelial cells, leukocytes*	+	*Lleukocytes*
Liver	22% (0.4) –	22% (0.4) 0% (0)	0% (0) 0% (0)	++	*Cholangiocytes,* *endothelial cells*	NE		−	*–*
Heart	50% (0.5) –	0% (0) 33% (0.3)	38% (0.4) 0% (0)	+	*Endothelial cells*	+	*Endothelial cells, mesothelial cells*	+	*Leukocytes*
Kidney	11% (0.1) –	0% (0) 0% (0)	0% (0) 0% (0)	++	*Endothelial cells*	NE		−	–
Brain	0% (0) –	67% (1.3) 57% (1.1)	14% (0.1) 0% (0)	++	*Endothelial cells*	++	*Neurons, glial cells, necrotic debris*	–	–
Pancreas	0% (0) –	0% (0) 0% (0)	0% (0) 0% (0)	+	*endothelial cells*	++	*Endothelial cells*	−	–
Proventriculus	0% (0) –	56% (0.7) 60% (1.0)	0% (0) 0% (0)	+	*endothelial cells*	++	*Endothelial cells*	NA	
Intestine	67% (1.0) –	67% (1.0) 71% (1.0)	0% (0) 50% (0.5)	++	*Endothelial cells, leukocytes, epithelium*	++	*Endothelial cells, leukocytes, (epithelium)*	+	*Epithelium, leukocyte*

*4-point scale Histopathological scoring (Additional file 1).

**Viral antigen immunohistochemical detection (Additional file 2).

NA: non available.

Pulmonary lesions were present in 100%, 89% and 25% of naturally dead guinea fowls, chickens and ducks, respectively. The severity score was significantly higher in guinea fowls and chickens compared to ducks (Figure 2). In Galliformes, lesions consisted in marked diffuse acute interstitial pneumonia with congestion, edema, heterophilic and lymphohistiocytic infiltration of air capillaries and interlobular spaces. Additionally, multifocal capillary thrombosis, single cells necrosis/apoptosis and parenchymal necrosis were observed in guinea fowls and chickens (Figures 3 and 4). Similarly to spleen, lymphocytolysis was frequently observed in chickens. In ducks, mild and non-specific findings, including congestion and lymphoplasmacytic infiltration, predominated. Mucosal epithelial degeneration and necrosis were occasionally observed in guinea fowls and a single duck (Figures 4 and 5). Nucleoprotein detection was widespread in guinea fowls, frequent but variable in chickens and localized mainly within the air capillaries and endothelial cells of interlobular blood vessels. In ducks nucleoprotein detection was rare and sparse, except for one duck exhibiting a more intense signal within multiple areas of bronchial necrosis.

Tracheal lesions in chickens involved mainly the lamina propria and included congestion, edema, and heterophilic infiltration, with endothelial expression of viral nucleoprotein. Similarly to chickens, acute vascular lesions and viral antigenic detection were observed in guinea fowls, together with cytopathic effects involving the respiratory epithelium, such as deciliation, vacuolar degeneration, single cell necrosis/apoptosis and necrosis. In ducks, non-specific lymphoplasmacytic infiltration was observed in the lamina propria with no evidence of nucleoprotein expression.

Brain lesions were remarkable in chickens, with multifocal random foci of neuronal necrosis and gliosis scattered within cerebral and cerebellar parenchyma. These lesions were detected in 67% and 57% of dead and euthanized birds, respectively, and were associated with NP-positive glial cells, neurons and necrotic debris (Figures 2 and 3). In ducks, focal lymphoplasmacytic perivascular cuffing was present in one subject (14%), with no positive NP detection. The nervous tissue was unremarkable in all dead guinea fowls, despite concurrent detection of NP within the endothelial cells.

The liver showed degeneration and necrosis of the biliary epithelium of the portal tracts in two guinea fowls (22%), with frequent NP-positive labelling of cholangiocytes and cellular debris (Figure 4). In all species, periportal mixed leukocytic infiltration, with no nucleoprotein colocalization, was frequently observed. This last change was interpreted as a background finding.

Despite the low frequency, heart lesions differed among the species examined. In chickens, 33% of euthanized subjects presented congestive and lymphohistiocytic epicarditis associated with NP-positive endothelial and mesenchymal cells. In 50% of guinea fowls, the myocardium was mildly congested and exhibited heterophilic leukostasis with sparse positive nucleoprotein detection in the endothelial cells. In ducks, mild, multifocal lymphocytic interstitial myocarditis was observed in 3 dead birds (33%). Sparse positive nucleoprotein detection was present in the interstitium, but complete cellular identification was not possible.

Proventricular lesions predominated in chickens (56–60%), compared to guinea fowls (0%) and ducks (0%). Lesions consisted in interstitial acute haemorrhages and single cell necrosis/apoptosis of the tip of the proventricular glands, in close proximity to the primary ducts. This observation was consistent with the macroscopic distribution pattern and was associated with endothelial nucleoprotein detection (Figure 3).

Kidney lesions were mostly non-specific and limited to diffuse congestion. Cecal sections showed single cell necrosis/apoptosis within the lamina propria in chickens (67–71%) and guinea fowls (67%), with prominent lymphocytolysis and macrophages containing tingible bodies. NP-positive cells were frequently observed within the lamina propria (endothelial cells and leukocytes) and epithelium (Figures 3 and 4). For ducks, non-specific lesions were found in the ceca. In one subject, an intestinal section weak NP-positivity was observed in one duck within intestinal mucosa.

Pancreas was within normal limits in all subjects. However, nucleoprotein was detected in the endothelial cells in the pancreas of guinea fowls and chickens.

Overall, these results suggest a strong lesional expression in infected Galliformes and a poor expression in ducks. In chickens, lesions were consistent with a systemic acute interstitial inflammation characterized by vasculitis, lymphocytolysis, and encephalitis. In Guinea fowls, lesions appeared similar to chickens in terms of endothelial involvement, while epithelial lesions were more common and necrotizing encephalitis was absent. In ducks, lesions were unfrequent and poorly specific, but indicative of a systemic infection and a significant cardiomyotropism in a few subjects.

### Viral RNA and antigenic quantification

**Figure 6:**
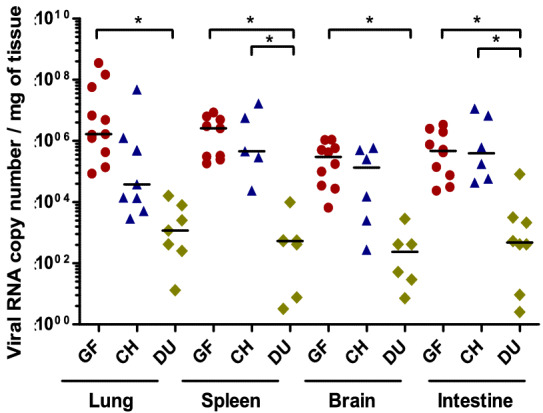
**Viral RNA tissue loads of naturally infected guinea fowls, chickens and ducks with H5 HPAIV.** For each organ (lung, spleen, brain, intestine) viral RNA loads were compared between species. * indicates statistical significance (*p* < 0.05, Kruskal–Wallis’ test with Dunn-Bonferroni post hoc test). Black lines represent medians of viral RNA load (Viral RNA copy number per mg of tissue) and each point represents subject that was found dead (CH, chicken; GF, guinea fowl; DU, duck).

**Figure 7:**
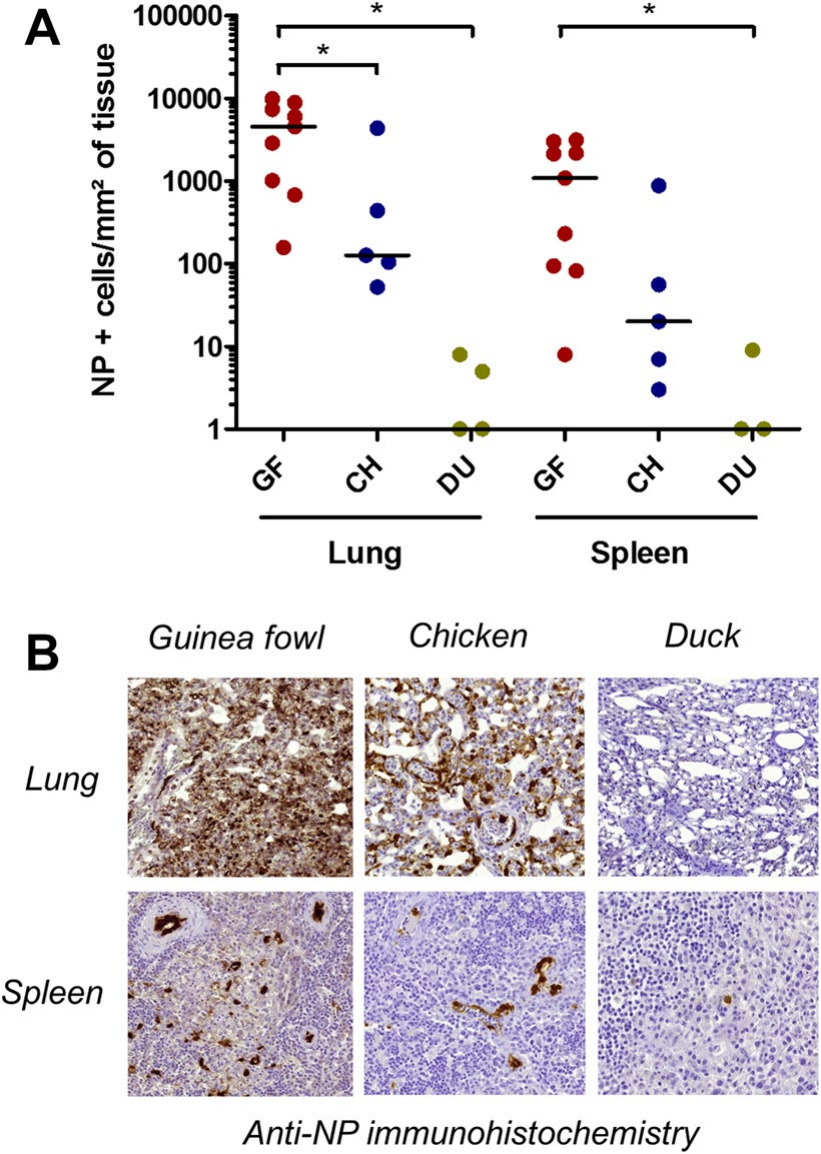
**Nucleoprotein-positive cells density within tissue sections (lung and spleen) of naturally infected guinea fowls, chickens and ducks with H5 HPAIV.** Anti-NP Immunohistochemistry. **A** Dot plot graph (CH, chicken; GF, guinea fowl; DU, duck). Black lines represent medians and each point represents a subject that was found dead. * indicates statistical significance (*p* < 0.05, Negative binomial regression. **B** Representative immunohistochemistry pictures.

Compared to dead ducks, viral RNA loads were significantly higher in dead guinea fowls for brain, lung, spleen and intestine and in dead chickens for spleen and intestine (*p* < 0.05, Figure 6). Nucleoprotein-positive cells density was significantly higher in guinea fowls compared to ducks for both spleen and lung and in chickens for lung (*p* < 0.05, Figure 7).

### Glycan and host tissue binding specificities of H5 HPAIV hemagglutinin

**Figure 8:**
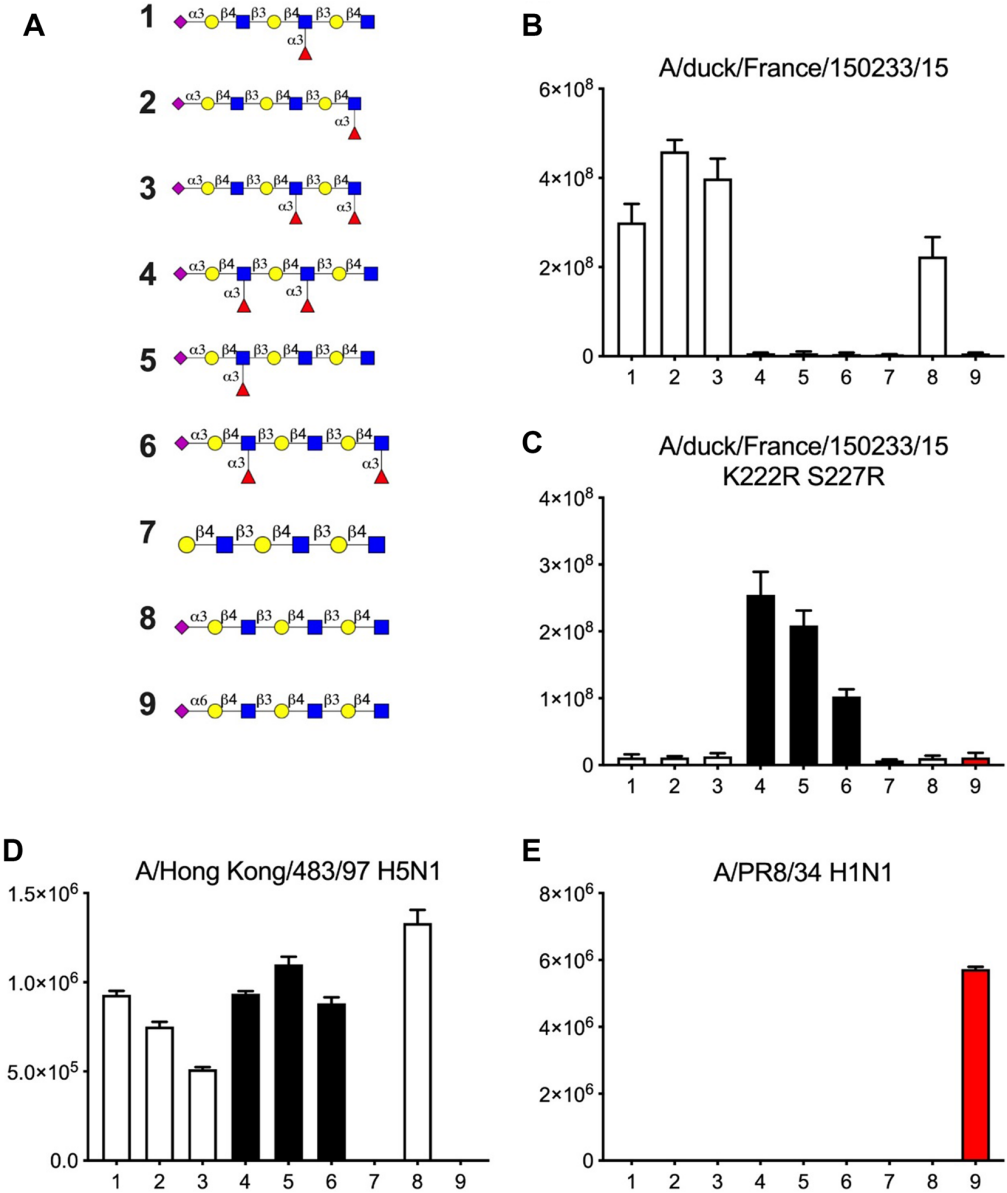
**Glycan binding profiles of wildtype and mutated H5 HPAIVs recombinant hemagglutinin using glycan microarray**. Fluorescence. **A** Glycans identification. Glycan profile of wiltype H5 HPAIV (**B**), mutated H5 HPAIVs (**C**) in comparison with H5N1 (**D**) and H1N1 (**E**). Bar graphs represent the averaged mean signal minus the background for each glycan sample, and error bars are the SD values.

Glycobiologic determinants of interspecie barrier were investigated using recombinant HA (rHA) generated from a H5 HPAIV isolated from an infected duck. On glycan array, recombinant wildtype H5 hemagglutinin preferentially bound sialylated LacNAc, but did not bind Sialyl-Lewis^X^**,** suggesting terminal fucose is not tolerated (Figure 8B). The introduction of K222R and S227R substitutions changed affinity towards Sialyl-Lewis^X^, suggesting the requirement of those two mutations for the binding of rHA to fucosylated SAs (Figure 8C). As controls we took along a recombinant H5 from the A/HongKong/483/98 strain that did not differentiate between sialylated LacNAc and sialyl-Lewis^X^ (Figure 8D) and a H1 from A/PR/8/34 known to bind α2,6 linked sialic acid and that, as expected, only showed responsiveness to structure #9 (Figure 8E).

**Figure 9:**
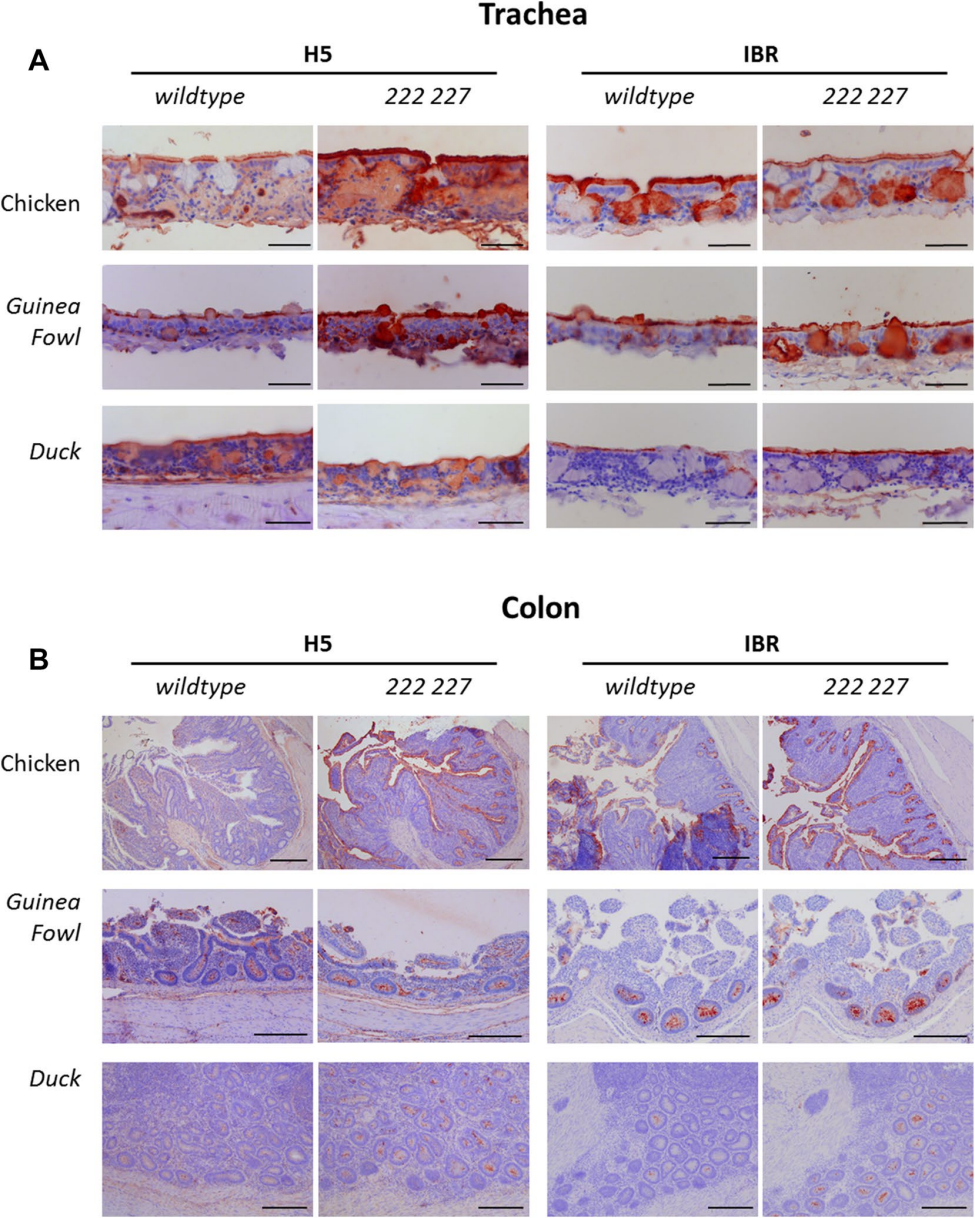
**Tissue binding profile of wildtype and mutated rHA on chicken, guinea fowl and duck mucosa.** Protein immunohistochemistry. Wildtype and mutated 222 227 rHA from A/duck/France/150236/15 (H5N9) and A/chicken/Ibaraki/1/2005 (IBR) were applied on tracheal and colon mucosa. rHA binding appears as brown on epithelium.

Affinity of H5 rHA for chicken, guinea fowl and duck mucosae was then determined on tissue microarrays including trachea and colon tissues, and compared with the chicken-adapted H5N2 strain A/chicken/Ibaraki/1/2005 (IBR, Figure 9). Wildtype H5 rHA binding was positively detected in tracheal epithelium of all three species. Both 222 and 227 mutations of H5 rHA increased mucosal affinity for trachea from chicken and guinea fowl. Affinity pattern was similarly observed on colon mucosa from duck and chicken. Interestingly, affinity pattern for guinea fowl colon was not dependent on 222 and 227 mutations and similar to duck. Conversely, Wildtype and mutated H5 (IBR) rHA revealed lower positive detection on duck mucosae compared to those of guinea fowls and chickens.

Overall, these results indicate that the hemagglutin from the H5 HPAIV isolated during the 2015–2016 epizootic presents a glycan-binding specificity consistent with duck-adapted AIVs, and suggest that the amino acids at positions 222 and 227 of the hemagglutinin sequence are major determinants of mucosal and glycan binding affinity for duck and chicken mucosae [12, 13]. Compared to duck and chicken, which represent two extremes in terms of host tissue affinity profiles, guinea fowl was found to be intermediate, sharing partially similar binding pattern with duck and chicken.

## Discussion

During winter 2015–2016, an epizootic involving several H5 HPAIVs was registered in commercial poultry flocks located in the South-West of France. This epidemic originated from the local emergence of an original H5 HPAIV, unrelated to the H5 HPAIV Gs/Gd lineage. The present work provides a unique pathobiological characterization of this epizootic in naturally infected ducks, guinea fowls and chickens, including the investigation of glycobiologic determinants contributing to species susceptibility.

HPAIVs display a tissular pantropism, resulting from the mutational acquisition of a multibasic sequence in the haemagglutinin cleavage site. Shifting from mucosal epitheliotropism to tissular pantropism leads to systemic disease and high mortality in gallinaceous species [3, 20]. On the contrary, clinical expression of HPAIV infection in ducks is generally poor, albeilt there have been increasing evidence of virulence and contagion in ducks infected with H5 HPAIVs Clade 2.3.4.4 in particular [21, 22]. As previously mentioned, species sensitivity and viral tropism are intimately linked to the interaction between viral and host molecular determinants, including cellular susceptibility, accessibility and permisiveness [7–9].

Innate response counteracting viral replication has been recently reviewed for chicken and duck [9]. Mule ducks are known to have a strong type I interferon (IFN)-mediated innate immune response**,** limiting viral replication in the days following infection, and contributing to low clinico-pathological expression. In our studies, lesions in ducks were relatively mild and viral RNA load relatively low. Non-suppurative myocarditis and encephalitis were observed, but this finding was limited to few subjects, in contrast with more recent outbreaks caused by clade 2.3.4.4b Gs/GD H5 HPAIVs in which neurotropism and cardiomyotropism led to increased mortality and lesions in ducks [22–24].

On the contrary, infection in chickens was mostly lethal and associated with severe systemic acute interstitial inflammatory lesions with evidence of both endotheliotropism and neurovirulence. Nervous lesions and distribution were consistent with previous spontaneous and experimental infections in chickens with other HPAIVs [25, 26] and could have presumably contributed to acute death in addition to systemic organ failure or cardiovascular damage. Studies suggested that chickens have a lower antiviral innate immune response compared to ducks. They lack RIG-I, a cellular sensor that detects 5’-triphosphorylated RNA and viral transcriptional intermediates of influenza virus, initiating IFN response [9, 27]. Such defective defense could potentially explain the lethality and strong lesional expression observed in this case.

For guinea fowls, clinicopathological manifestations were similar to chickens. Strinkingly, lesions and both viral antigenic and RNA detections appeared substantially higher and less variable than chickens and viral immunohistochemical antigen detection was in favor of both endotheliotropism and epitheliotropism. Additionally, haemagglutinin binding profile demonstrated intermediate affinity between duck and chicken profiles. Altogether, these results suggest that guinea fowls could be more susceptible to infection with these H5 HPAIVs than chickens but similarly permissive. Strong susceptibility of guinea fowls was reported with asian 2.3.4.4a Gs/GD H5 HPAIV [28]. In that study, compared to others galliforms (ie. chicken, quail, partridge, pheasant), guinea fowls presented the lowest mean bird infectious dose 50 and the shortest mean death time [28]. Further investigations in experimental settings and on a wide range of HPAIVs are needed to ascertain the susceptibility of guinea fowls, and their possible role in the transmission of HPAIVs from ducks to chickens.

Despite the evidence of pathobiological differences between the species involved in this study, several factors could impact clinico-pathological and scoring results, limiting comparison. Our infected animals were the result of an epizootic involving several H5 HPAIVs reassortants. Phylogenetic analyses conducted on seven isolates revealed a very close proximity between hemagglutinin sequences, confirming that these viruses are part of the same epizootic cluster. Genetic reassortments have been suggested for neuraminidase and other viral segments, as classically observed during HPAI epizootics [4]. Consequently, the contribution of such genetic constellations in the clinico-pathological differences observed in the present study cannot be ruled out and would require further investigations, with one viral genotype inoculated to different bird species in controlled experimental conditions. The second potentially confusing factor involves the stage of infection that could differs between subjects, flocks and species. Our data originate from naturally infected flocks that may have been analyzed at different timepoints of infection during the progression of the epizootic. However, birds were studied separately according to the clinical stage.

Receptor-dependent entry into host cells is a critical determinant of viral tropism. AIVs are known to preferentially bind cells on α2–3 linked N-acetylneuraminic sialic acid residues of glycoproteins through the hemagglutin protein. Among avian species, AIV hemagglutinin contributes to species restriction: duck-adapted AIVs preferentially bind 3’ Sialyl-LacNAc whereas chicken-adapted AIVs prefrentially bind Sialyl-Lewis^X^ which is abundant in chicken trachea but undetected in duck colon [29]. Previous studies on Eurasian H5 AIVs suggested that specific mutations at the 222 and 227 amino acid positions in the hemagglutinin protein are critical to shift specificity from 3’ Sialyl-LacNAc to Sialyl-Lewis^X^ abundant in chicken trachea [12, 13]. This is suggested to be an adaptative process, occurring in infected Galliformes, through replicative mutagenesis and promoting adaptation and selection of chicken-adapted viruses [30]. In our case, the isolated H5 HA presented low affinity for Sialyl-Lewis^X^ with similar-receptor binding domain compared to A/Duck/Mongolia/54/2001 (H5N2). An increase in the adhesion of HA to chicken trachea and Sialyl-Lewis^X^ was observed when both K222R and S227R mutations were introduced into the HA sequence. Subsequently, infection of chicken in our field cases could presumably be explained by some additional factors pressuring the species barrier and promoting viral penetration to susceptible structures such as the endothelium. Suitable factors, contributing to mucosal crossing, could include: exposure to high viral loads, polymicrobial infection, high density promoting close and prolonged contact between chickens, guinea fowls and duck or other environmental and management parameters.

To conclude, the present study provides a pathobiological comparison of Galliformes and Anseriformes naturally infected with H5 HPAIVs involved in an HPAI epizootic registered in France during winter 2015–2016. The isolated H5 HPAIVs appeared adapted to ducks based on glycan and mucosal binding affinity profiles. In Galliformes, infection resulted in severe and systemic disease, but clinical expression varied between flocks, suggesting the potential role of additional factors promoting the cross of species barrier in chickens. Interestingly, guinea fowls showed pathobiological findings that appeared to be a combination of the ones observed in chickens and ducks. Further studies are needed to confirm the susceptibility of guinea fowls to HPAIV’s and their role in viral transmission.

## Supplementary Information

**Additional file 1. Epidemio-clinical characteristics of flocks included in the study.**

**Additional file 2. Phylogenetic tree of the H5 gene sequences, including 7 H5 avian influenza viruses isolated from chicken, duck, guinea fowls in France, 2015–2016.**

**Additional file 3. Histopathological scoring system.**

**Additional file 4. Viral immunohistochemical antigenic detection scoring system.**

## Data Availability

The datasets used and/or analysed during the current study are available from the corresponding author.
