# Supplementary material for: Pathobiology of highly pathogenic H5 avian influenza viruses in naturally infected Galliformes and Anseriformes in France during winter 2015–2016
Source: Vet Res. 2022 Feb 14;53:11. doi: 10.1186/s13567-022-01028-x (PMC8842868; doi:10.1186/s13567-022-01028-x)
Supplement: Supplementary file 1 — Additional file 1. Epidemio-clinical characteristics of flocks included in the study. [file 13567_2022_1028_MOESM1_ESM.docx]

**Additional file 1. Epidemio-clinical characteristics of flocks included in the study.**

| **Specie** | **Flock** | **Subtype** | **Date** | **Location** | **Clinical observation** | **Clinical status and number of sampled animals** |
| --- | --- | --- | --- | --- | --- | --- |
| Guinea Fowl | #1 | H5N9 HP | 06/12/2015 | Landes | Increased mortality (18%), facial edema | Dead (*n*= 4) |
|  | #2 | H5N9 HP | 09/12/2015 | Landes | Increased mortality (10%), facial edema. | Dead (*n* = 5) |
|  | #3 | H5N2 HP | 15/12/2015 | Gers | Increased mortality (7%), Prostration, facial edema (5%) | N/A |
| Chicken | #1 | H5 HP | 19/12/2015 | Gers | Increased mortality (N/A), facial edema and cutaneous hemorrhages | Euthanized (*n* = 2) Dead (*n* = 4) |
|  | #2 | H5 HP | 30/12/2015 | Landes | Increased mortality (N/A), facial edema, cutaneous hemorrhages and necrosis | Dead (*n* = 5) |
|  | #3 | H5N1 HP | 12/01/2016 | Haute-Garonne | Increased mortality (5-10%), respiratory distress, protrastion | Euthanized (*n* = 5) |
|  | #4 | H5 HP | 09/02/2016 | Pyrénées-atlantiques | Facial edema, cutaneous hemorrhages and necrosis | N/A |
| Duck | #1 | H5N9 HP | 10/12/2015 | Pyrénées-atlantiques | Mild mortality, few individuals with dyspnea, one with nystagmus and tremor | Euthanized (*n* = 2) Dead (*n* = 3) |
|  | #2 | H5 HP | 19/12/2015 | Pyrénées-atlantiques | Mild mortality | Dead (*n* = 5) |

N/A not available.
