# Supplementary material for: Pathobiology of highly pathogenic H5 avian influenza viruses in naturally infected Galliformes and Anseriformes in France during winter 2015–2016
Source: Vet Res. 2022 Feb 14;53:11. doi: 10.1186/s13567-022-01028-x (PMC8842868; doi:10.1186/s13567-022-01028-x)

**Additional file 2. Phylogenetic tree of the H5 gene sequences, including 7 H5 avian influenza viruses isolated from chicken, duck, guinea fowls in France, 2015-2016.**


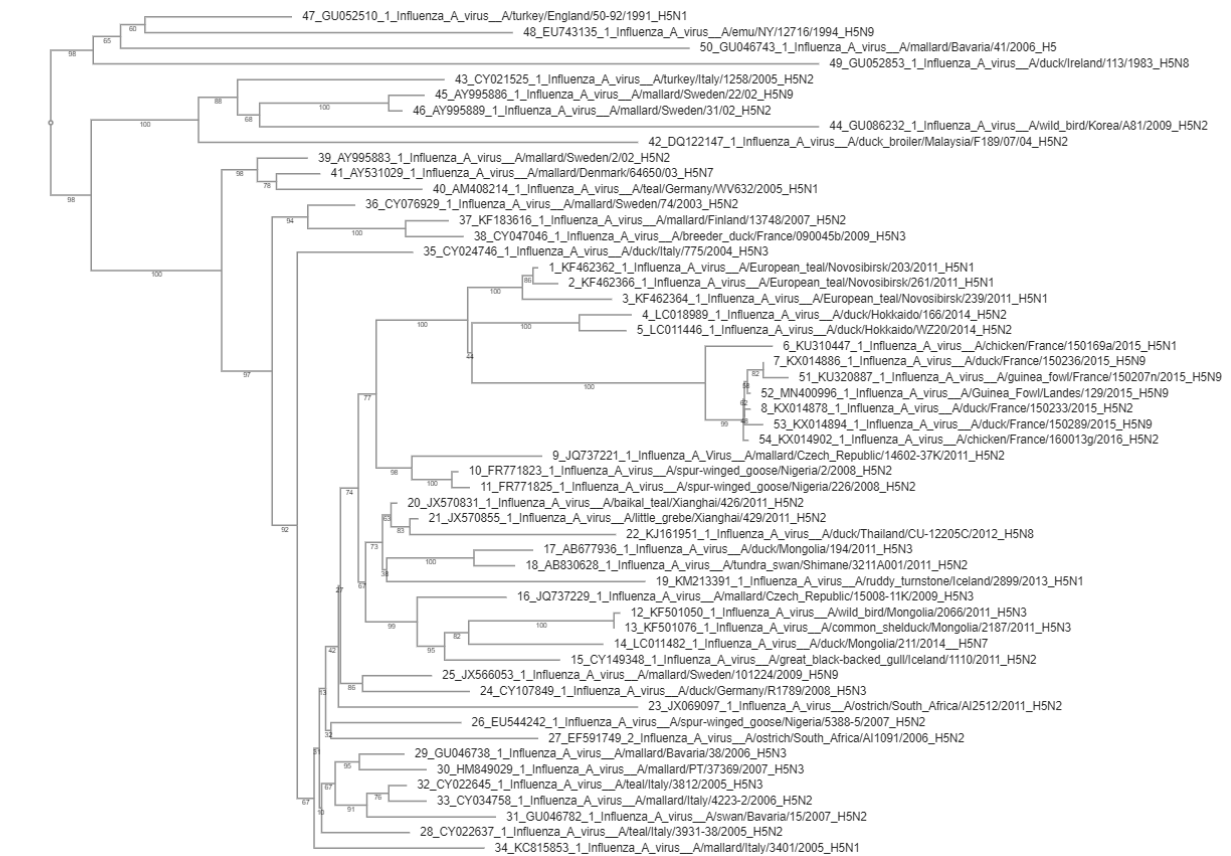

Supplement: Supplementary file 2 — Additional file 2. Phylogenetic tree of the H5 gene sequences, including 7 H5 avian influenza viruses isolated from chicken, duck, guinea fowls in France, 2015–2016. [file 13567_2022_1028_MOESM2_ESM.docx]
