# Supplementary material for: Pathobiology of highly pathogenic H5 avian influenza viruses in naturally infected Galliformes and Anseriformes in France during winter 2015–2016
Source: Vet Res. 2022 Feb 14;53:11. doi: 10.1186/s13567-022-01028-x (PMC8842868; doi:10.1186/s13567-022-01028-x)
Supplement: Supplementary file 3 — Additional file 3. Histopathological scoring system. [file 13567_2022_1028_MOESM3_ESM.docx]

**Additional file 3. Histopathological Scoring System**

| **Score** | **Criteria** |
| --- | --- |
| 0 | Within normal limits |
| 1 | **Leukocytic interstitial infiltration. Absence of cytopathic effects*** |
| 2 | **Leukocytic interstitial infiltration with multifocal non coalescing lesions of parenchymal necrosis, single cell necrosis/apoptosis, vasculitis.** |
| 3 | **Leukocytic interstitial infiltration with multifocal coalescing lesions of parenchymal necrosis, single cell necrosis/apoptosis, vasculitis**. |

*4-point scaled scoring system based on nominal criteria defining lesion.***** Histological scoring of spleen is interpreted as 1, when splenic tissue exhibits signs of reactivity including expanded hyperplastic reticular cell, periarteriolar lymphoid sheaths and follicular hyperplasia****.*
