# Supplementary material for: Pathobiology of highly pathogenic H5 avian influenza viruses in naturally infected Galliformes and Anseriformes in France during winter 2015–2016
Source: Vet Res. 2022 Feb 14;53:11. doi: 10.1186/s13567-022-01028-x (PMC8842868; doi:10.1186/s13567-022-01028-x)
Supplement: Supplementary file 4 — Additional file 4. Viral immunohistochemical antigenic detection scoring system. [file 13567_2022_1028_MOESM4_ESM.docx]

**Additional file 4. Viral immunohistochemical antigenic detection scoring system.**

| **Score** | **Criteria** |
| --- | --- |
| **-** | None |
| **+** | Sparse |
| **++** | Frequent |
| **+++** | Widespread |

**
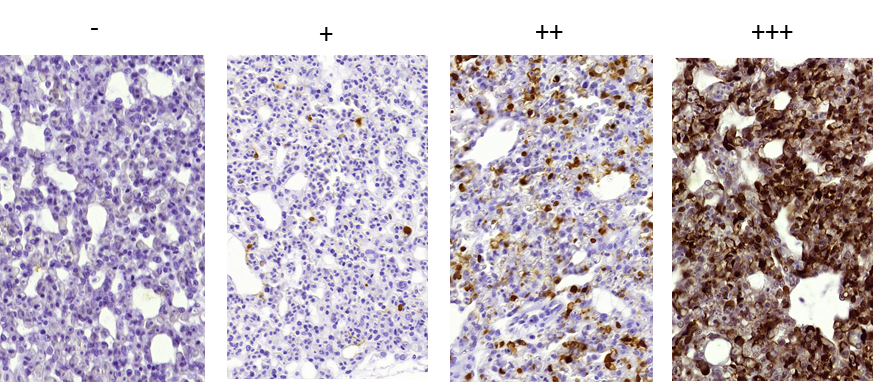
**
